# Using the Game of Mastermind to Teach, Practice, and Discuss Scientific Reasoning Skills

**DOI:** 10.1371/journal.pbio.1000578

**Published:** 2011-01-18

**Authors:** Amy R. Strom, Scott Barolo

**Affiliations:** Department of Cell and Developmental Biology, University of Michigan Medical School, Ann Arbor, Michigan, United States of America; University of California Berkeley/Joint Genome Institute, United States of America

## Abstract

The code-breaking game Mastermind, which can be played in minutes at no cost, creates opportunities for students to discuss scientific reasoning, hypothesis-testing, effective experimental design, and sound interpretation of results.

In order to develop into effective researchers, educators, and science professionals, students must internalize basic principles of scientific reasoning and experimental design. Scientific reasoning skills can improve with training [1], but they can be difficult to impart as abstract concepts in the classroom. Here, we discuss the potential for using the game of Mastermind as a tool to help students develop logic skills, design effective experiments, and discuss scientific reasoning in the classroom or lab.

The English code-breaking game known as Bulls and Cows, popularized as the board game Mastermind, has been adapted for applications in fields such as mathematics, computing, and psychology [2]–[9]. Mastermind has been proposed as a tool for teaching logic in mathematics courses [10], but the problem-solving skills emphasized by the game are also relevant to the life sciences. We propose that the game can be used to teach specific lessons and spark discussions about scientific reasoning, covering topics such as sound experimental design, hypothesis-testing, careful interpretation of results, and the effective use of controls.

In certain respects, the game simulates an experimental research project, but it can be played in minutes, at no cost. Advanced language skills, prior scientific training, and lab facilities are not required. See Text S1 for a full explanation of the rules of the game. Briefly, the “codemaker” creates a secret code, which the “codebreaker” attempts to discover in as few turns as possible. In the examples provided here, the code is an ordered sequence of four colors, selected from six possible colors: Red, Blue, Green, Yellow, Orange, and Pink. The codebreaker takes a guess at the code (i.e., performs an experiment), interprets the feedback provided by the codemaker (i.e., the result of the experiment), and uses this information to design the next experiment. Because winning depends on reducing the 6^4^ = 1,296 possible solutions to one in the fewest possible experiments, sound logical reasoning and good experimental design are essential. The game therefore provides a simple, practical framework for the discussion and practice of important scientific skills. It's also fun to play. In this report, we will describe opportunities for lessons and discussions concerning scientific reasoning that often occur during gameplay. We will also present ideas for adapting the game as a classroom or lab exercise.

## Logical Reasoning in Mastermind: An Annotated Sample Game

**Figure 1:**
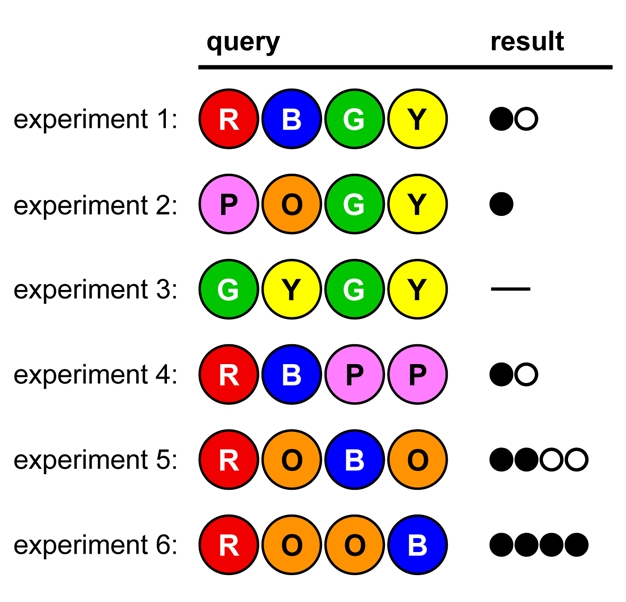
A sample game. See text for conclusions following from each result.

**Figure 1** shows a game (adapted from actual mentor-student games) in which the codebreaker's conclusions are written out, to demonstrate how logic is used to break the code. In the codemaker's feedback, a black dot indicates a correct color in the correct position; white indicates a correct color in the wrong position (see Text S1 for full rules; see Text S2 for a fully annotated diagram of the sample game).

The codebreaker can draw the following conclusions during the game:

*Conclusions from Experiments 1 and 2:*

- All colors have been tested and only three dots have been given in total; therefore at least one color is repeated in the code.
*Conclusions from Experiment 3:*
- The code has no Green or Yellow.
- Therefore, the code has both Red and Blue, and either Pink or Orange (but not both).
- Based on experiment 1, either Red is in first position and Blue is in third or fourth, or Blue is in second and Red is in third or fourth.
- Based on experiment 2, either Pink is in first position or Orange is in second.
*Conclusions from Experiment 4:*
- The code has no Pink. The code contains only Red, Blue, and Orange.
- Following conclusion *v*, Orange must be in second position.
- If Orange is in second position, Blue can't be. Following conclusion *iv*, Red must therefore be in first. The only codes consistent with these data are ROBB, ROBO, ROBR, ROOB, and RORB.
*Conclusions from Experiment 5:*
- Orange is repeated.
- Blue must be in fourth position. Only one possible code remains.

The code is broken on the sixth attempt, so the codemaker receives six points.

## Five Teachable Moments during Gameplay

The following examples of situations that commonly occur in games of Mastermind, adapted from actual student games, present opportunities for discussions about scientific reasoning and experimental design.

### Lesson 1: Well-controlled experiments allow strong, specific conclusions

**Figure 2:**
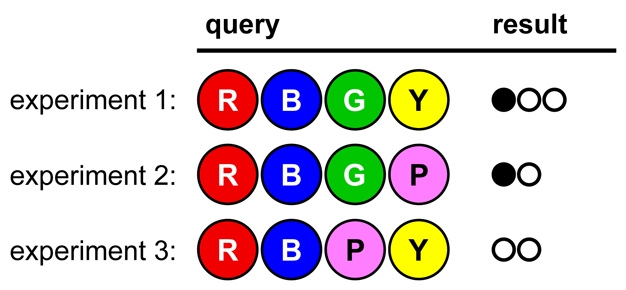
A game opening illustrating Lesson 1.

In the game shown in **Figure 2**, notice how only one color is changed in each successive experiment:

Comparing the first two results, we conclude that Yellow is in the code (but not in fourth position) and Pink is out, since replacing Yellow with Pink resulted in the loss of a white dot. Similarly, comparing result 3 with result 1, we see that Green must be in third position, since removing Green causes the loss of a black dot. We can further deduce that the code contains Red (in second and/or fourth position) or Blue (in first and/or fourth position), but not both. Because each experiment serves as a useful comparison for the others, we have reduced the viable hypotheses from 1,296 to 18 in only three tries.

Ask students to list the viable hypotheses and to design a follow-up experiment that will eliminate at least half of the remaining possible solutions.

### Lesson 2: Over-interpretation of data leads to false conclusions

**Figure 3:**
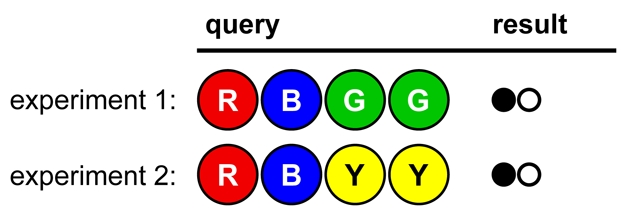
A game opening illustrating Lesson 2.

**Figure 4:**
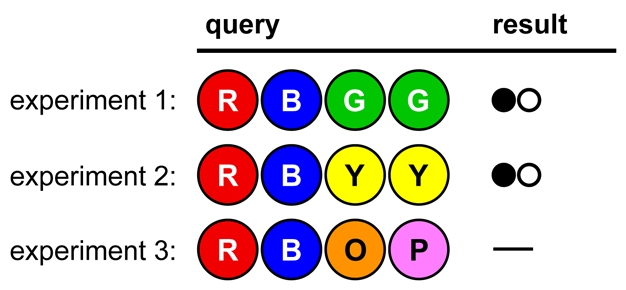
Continuation of the game from **Figure 3**.

Consider the game shown in **Figure 3**:

The codebreaker may conclude, “Changing the Greens to Yellows didn't change the result; therefore Red and Blue must be in the code, and Green and Yellow are out.” If so, the next result (**Figure 4**) will be a surprise:

The codebreaker made an unsafe assumption; in fact, the code is **YGYG**. This is an opportunity to point out that all reasonable interpretations that are consistent with the data must be considered. Ask students: What *exactly* does that result tell you? What is strictly ruled in or ruled out? Can you now get more information from previous experiments?

### Lesson 3: The value of negative data

**Figure 5:**
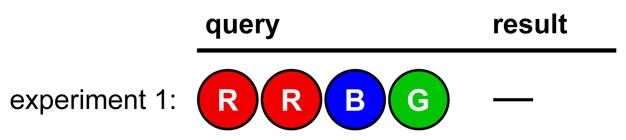
A game opening illustrating Lesson 3.

Consider the opening move in **Figure 5**:

A novice codebreaker may say “Bad luck! I struck out.” This presents an opportunity to point out the value of negative results: they are wonderful for invalidating hypotheses. Once students see that 94% of the possible solutions have been eliminated with one experiment, they may appreciate the usefulness of “striking out.” Ask students to find an example of an informative negative result in a research article.

### Lesson 4: Good experimental design saves time in the long run

**Figure 6:**
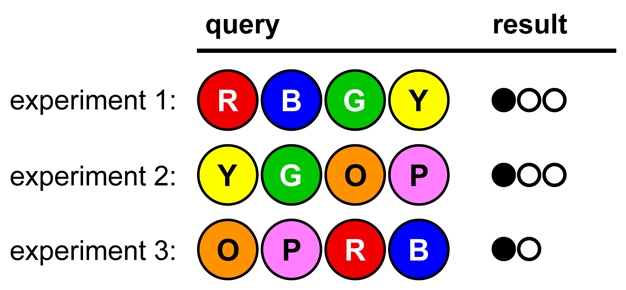
Opening strategy A, illustrating Lesson 4.

Compare the following three opening strategies:

In **Figure 6** we see a poorly designed, poorly controlled set of experiments. No firm conclusions can be drawn, because too many variables have changed in each experiment. For instance, no colors can be ruled in or out.

**Figure 7:**
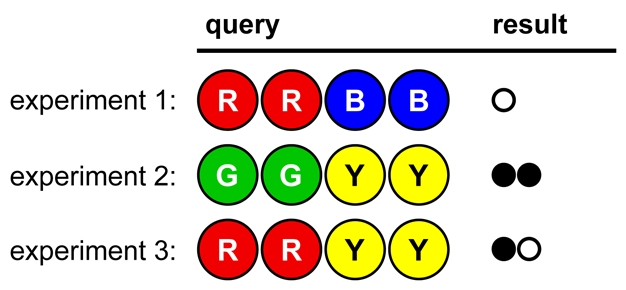
Opening strategy B, illustrating Lesson 4.

In **Figure 7**, fewer colors are tested per experiment, and no colors have changed position. This allows firm conclusions to be drawn from the results, reducing the number of possible solutions to eight. (The remaining viable hypotheses are GORY, GOYR, GPRY, GPYR, OGRY, OGYR, PGRY, and PGYR.)

**Figure 8:**
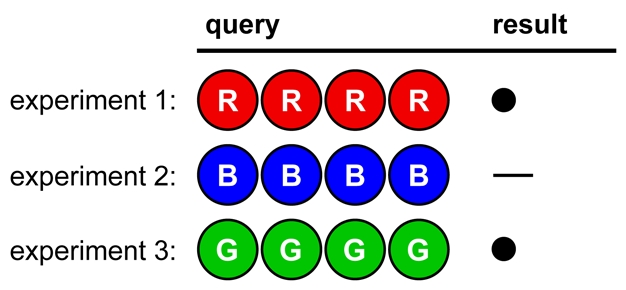
Opening strategy C, illustrating Lesson 4.

In **Figure 8**, the codebreaker's approach is admirably systematic, but inefficient [2]. Up to five experiments will be needed just to test all of the colors, with nothing learned about position.

The advantages and disadvantages of different approaches will naturally emerge in discussion among teammates, leading to debates about the best experimental design in a given situation.

### Lesson 5: Rather than seeking to confirm your hypothesis, test it as severely as possible. If a hypothesis is invalid, discard it immediately

We begin each game with 1,296 plausible models, and our goal is to invalidate 1,295 of them as quickly as possible. *Confirmation bias*—favoring one interpretation in the absence of evidence, or in the face of contradictory evidence [11],[12]—leads to unsound assumptions and wasted time and effort. We make no claims about the effectiveness of Mastermind in “correcting” confirmation bias, but the game at least presents opportunities to discuss this important issue, and demonstrates that when evidence contradicts a hypothesis, one must abandon or modify the hypothesis and move forward.

## Implementation in the Classroom or Lab

Mastermind can be played at little or no cost on a whiteboard, notebook, or a purchased or improvised game board. Any kind of symbols can be used, including colors, letters, numerals, shapes, or objects such as colored thumbtacks; the Mastermind family of board games uses colored pegs. Colors make the game more visually interesting and may engage visual learners, but using letters or numerals allows the game to be played remotely by web or email, and simplifies assigning specific game problems to students. The Advanced Mastermind board game (made by Pressman Toys in the USA) uses eight colors and a code length of five, but fewer colors and shorter codes can be used on the same board, allowing a wide range of difficulty.

Mastermind is traditionally a two-player game, but we find that it works well as a team exercise, in which students collaborate to break the code. Encourage students to talk through the logic underlying their conclusions, and to debate strategies for the next experiment. In our experience with playing against a mixed class of graduate students and undergraduates, and with casual games in the lab, some students will strongly advocate certain hypotheses, and others will look for flaws in their reasoning. The instructor can comment on students' logic without giving away the code. However, if you disagree with a student's strategy, it can be more instructive to let it fail, rather than shooting it down. Of course, this approach is often impractical with real lab experiments.

We ask those who try Mastermind in science teaching to post comments to this article. We especially encourage new ideas for adapting the game to the classroom or lab, new teachable moments, and evaluations of the game's instructional value.

## Supporting Information

Text S1 **Rules of the game of Mastermind.**
(0.06 MB PDF)

Text S2 **An annotated sample game.**
(0.17 MB PDF)
