# Supplementary material for: Using the Game of Mastermind to Teach, Practice, and Discuss Scientific Reasoning Skills
Source: PLoS Biol. 2011 Jan 18;9(1):e1000578. doi: 10.1371/journal.pbio.1000578 (PMC3022521; doi:10.1371/journal.pbio.1000578)
Supplement: Text S1 — Rules of the game of Mastermind. (0.06 MB PDF) [file pbio.1000578.s001.pdf]

Supporting Information

PLoS Biology: "Using the Game of Mastermind to Teach, Practice, and Discuss Scientific Reasoning Skills," by Amy R. Strom & Scott Barolo.

Text S1: Rules of the Game of Mastermind

One player takes the role of "codemaker" and creates a secret code, an ordered sequence of four colors. In the standard version of the game, the codemaker chooses from six colors—for example, RED, BLUE, GREEN, YELLOW, ORANGE, and PINK. Repeating colors in the code is typically allowed. The length of the code and the number of colors can be adjusted to make the game more or less challenging. The "codebreaker," who may be one person, a small team, or an entire class of students, must discover the secret code in as few attempts ("experiments") as possible. The codemaker receives points equal to the number of experiments required to break his or her code. The codemaker and codebreaker then switch roles, a second game is played, and the side with more points is the winner.

Each game proceeds as a series of experiments: the codebreaker presents a *query*, consisting of a guess about the code, such as RRGO (RED, RED, GREEN, ORANGE, in that order). The codemaker then gives a *result* that describes the accuracy of the query, using a black dot for each correct color in the correct position, and a white dot for each correct color in the wrong position. The order of the dots in the result does not reflect the order of colors in the query: black dots are given first, then white. (Importantly, if a repeated color in the query matches a non-repeated color in the code, only one dot is given; if one of the repeats is in the correct position, the dot is black.) For example, if the code is **RYYG**, a query of RRGO would produce a result of one black dot and one white dot. Experiments continue until the query matches the code.
