# Supplementary material for: Using the Game of Mastermind to Teach, Practice, and Discuss Scientific Reasoning Skills
Source: PLoS Biol. 2011 Jan 18;9(1):e1000578. doi: 10.1371/journal.pbio.1000578 (PMC3022521; doi:10.1371/journal.pbio.1000578)
Supplement: Text S2 — An annotated sample game. (0.17 MB PDF) [file pbio.1000578.s002.pdf]

Supporting Information

PLoS Biology: "Using the Game of Mastermind to Teach, Practice, and Discuss Scientific Reasoning Skills," by Amy R. Strom & Scott Barolo.

Text S2: An Annotated Sample Game

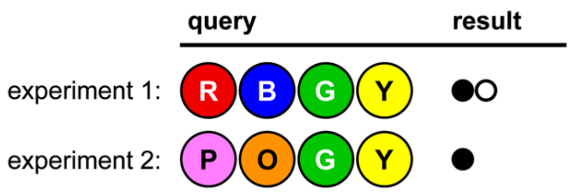

Conclusions:

i. All colors have been tested and only three dots have been given in total; therefore at least one color is repeated in the code.

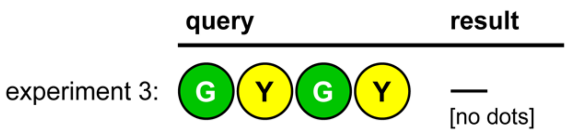

Conclusions:

ii. The code has no GREEN or YELLOW.

iii. Therefore, the code has both RED and BLUE, and either PINK or ORANGE (but not both).

iv. Based on experiment 1, either RED is in first position and BLUE is in third or fourth, or BLUE is in second and RED is in third or fourth.

v. Based on experiment 2, either PINK is in first position or ORANGE is in second.

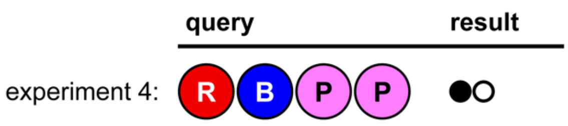

Conclusions:

vi. The code has no PINK. The code contains only RED, BLUE, and ORANGE.

vii. Following conclusion *v*, ORANGE must be in second position.

viii. If ORANGE is in second position, BLUE can't be. Following conclusion *iv*, RED must therefore be in first. The only codes consistent with these data are ROBB, ROBO, ROBR, ROOB, and RORB.

	query	result
experiment 5:	R O B O	●●○○

Conclusions:

ix. ORANGE is repeated.

x. BLUE must be in fourth position. Only one possible code remains.

	query	result
experiment 6:	R O O B	●●●●

(Codemaker gets 6 points.)
